# Detecting individual extracellular vesicles using a multicolor *in situ* proximity ligation assay with flow cytometric readout

**DOI:** 10.1038/srep34358

**Published:** 2016-09-29

**Authors:** Liza Löf, Tonge Ebai, Louise Dubois, Lotta Wik, K. Göran Ronquist, Olivia Nolander, Emma Lundin, Ola Söderberg, Ulf Landegren, Masood Kamali-Moghaddam

**Affiliations:** 1 Department of Immunology, Genetics & Pathology, Science for Life Laboratory, Uppsala University, SE-751 08 Uppsala, Sweden; 2 Department of Medical Sciences, Clinical Chemistry, Uppsala University, SE-751 85 Uppsala, Sweden

## Abstract

Flow cytometry is a powerful method for quantitative and qualitative analysis of individual cells. However, flow cytometric analysis of extracellular vesicles (EVs), and the proteins present on their surfaces has been hampered by the small size of the EVs – in particular for the smallest EVs, which can be as little as 40 nm in diameter, the limited number of antigens present, and their low refractive index. We addressed these limitations for detection and characterization of EV by flow cytometry through the use of multiplex and multicolor *in situ* proximity ligation assays (*in situ* PLA), allowing each detected EV to be easily recorded over background noise using a conventional flow cytometer. By targeting sets of proteins on the surface that are specific for distinct classes of EVs, the method allows for selective recognition of populations of EVs in samples containing more than one type of EVs. The method presented herein opens up for analyses of EVs using flow cytometry for their characterization and quantification.

Cells have the capacity to release different kinds of vesicles into the extracellular space, collectively named extracellular vesicles (EVs). EVs can be grossly subdivided into three subclasses based on their biogenesis and in order of increasing sizes; exosomes, microvesicles, and apoptotic bodies. Exosomes (including prostasomes derived from epithelial cells of the prostate gland^1^) are the smallest subclass, typically ranging in size between 40–100 nm in diameter, although larger sizes have been reported^1^,^2^,^3^. They arise through two invagination events; a first invagination of the plasma membrane produces “early endosomes” that mature into “late endosomes.” The latter undergo multiple invaginations, creating a multivesicular body whose membrane fuses with the plasma membrane from the inside, releasing its exosomal content into the extracellular space through exocytosis^2^,^3^. Microvesicles have sizes of 200–1,000 nm, and are the result of shedding from the plasma membrane – hence they are also denoted “shedding vesicles”^4^. Apoptotic bodies are the largest extracellular vesicles released during apoptosis and with diameters from one to a few μm^2^,^5^. EVs may exert pleiotropic biological functions by trafficking between cells. They can influence the microenvironment by transporting bioactive molecules, including proteins, lipids and RNA^6^,^7^,^8^. EVs have been implicated in physiological and pathological processes such as inflammation, immune disorders, and cancer^9^,^10^. Prostasomes have been found to be elevated in blood plasma from prostate cancer patients, where the levels of prostasomes in blood plasma may reflect the aggressiveness of the disease^11^.

Among the proteins most frequently identified in EVs are the tetraspanin family members CD9, CD63, and CD81, the small actin-binding protein cofilin-1, the heat shock proteins Hsp70, Hsp90, and enzymes involved in energy metabolism such as enolase, aldolase A, phosphoglycerate kinase 1 and glyceraldehyde 3-phosphate dehydrogenase^12^. A number of methods are currently available to detect and measure intact EVs. Nanoparticle tracking analysis (NTA) measures EV concentration and size based on scattered light or fluorescence (Malvern Instruments Ltd. United Kingdom). Resistive pulse sensing (RPS) determines the absolute sizes of the EVs in suspension using a specialized instrument, qNano, (Izon Science Ltd. New Zealand). A common limitation here is clogging of the system^13^. Enzyme-linked immunosorbent assays (ELISA) are used to analyze the proteins in a sample and are conventionally used to analyze EVs in clinical settings. This method, however, requires large amounts of EVs and is hampered by the time-consuming isolation process^13^,^14^. 4PLA developed in our lab is another sensitive method for detection of intact EVs. 4PLA requires binding by a total of five different antibodies in order to produce detectable signals, ensuring high specificity^11^. The PLA technology has been used to sensitively detect complex targets in body fluids, also in the presence of high concentrations of proteins in monomeric forms. EVs (prostasomes) were detected in the presence of abundant free protein molecules in plasma^11^ and amyloid beta protofibrils could be sensitively measured in cerebrospinal fluid despite a large molar excess of free amyloid beta monomers^15^. In 2014 Im *et al*. described the nano plasmonic exosome (nPLEX) assay, a label-free quantitative assay for analysis of exosomes. The assay is based on nanohole arrays, which can be utilized to profile the proteins on the surface of exosomes or in exosome lysates^16^.

EVs are also investigated for their contents, and many assays exist that are devoted to study e.g. mRNAs and miRNA transported in the vesicles, but these methods are beyond the scope of the present investigation.

Despite the plethora of techniques to find and characterize EVs in biofluids the need remains for improved methods. Flow cytometry-based methods are attractive for detecting EVs, both for qualitative and quantitative characterization. However, the small size of the EVs represents a considerable challenge, making individual EVs difficult or impossible to distinguish from background by conventional flow cytometry where the lower size detection threshold is around 500 nm^17^. Another problem is the paucity of antigen molecules present on the surface of the EVs due to their limited size, rendering detection with fluorophore-coupled antibodies very challenging. These difficulties are often resolved by using bead-based assays, or by lipid-membrane staining and bulk measurements^18^. In bead-based assays, EVs are captured on beads, and stained for surface markers using fluorophore-coupled antibodies. This is a satisfactory alternative for detecting the presence of EVs in a sample, since each bead can bind several EVs and stain for surface antigens, thus providing sufficiently strong signals to be detected in the flow cytometer. However, the current bead-based assays are not suitable for identification of individual EVs since the beads may carry a large number of EV particles sharing one antigen recognized by the capturing antibody.

To address limitations of flow cytometric detection of EVs, we applied the *in situ* proximity ligation assay (*in situ* PLA)^19^,^20^ in a method we refer to as ExoPLA. By using multicolor *in situ* PLA^21^ we label the EVs in three colors, in assays that depend on binding of individual EVs by a total of five different antibodies, providing outstanding assay specificity. Utilizing the signal amplification feature of *in situ* PLA, the signal from individual EVs is sufficiently strong to be detected well above the background. In this method EVs are captured on beads, followed by specific detection, washes, and then release from the beads before signal amplification via rolling circle amplification (RCA)^19^. The release of the EVs from the beads prior to signal amplification and detection serves to permit analysis of individual EVs in a flow cytometer, and to avoid the co-localization of signals generated from different EV on same bead particle.

*In situ* PLA utilizes PLA probes; antibodies coupled to DNA oligonucleotides. When two PLA probes are brought in proximity, a DNA circle can be obtained through two ligation reactions, templated by the antibody-bound oligonucleotides. This DNA circle then serves as a template for RCA. Detection oligonucleotides coupled to fluorophores are hybridized to the growing RCA product. Each detected protein gives rise to an RCA product with several hundred fluorophores, ensuring strong detection signals for individual EVs.

Since EVs from different cell sources may reflect the identity of the originating cell^22^, a method to distinguish different populations of EVs using conventional flow cytometry may permit enhanced resolution through analysis of individual EVs. As previously shown, prostasomes represent promising biomarker candidates in prostate cancer^11^. To test if ExoPLA is sensitive and specific enough to find prostasomes in a complex matrix, we spiked prostasomes isolated from seminal fluid in female blood plasma, demonstrating the ability of ExoPLA to accurately detect prostasomes against a background of all resident proteins and EVs in female plasma.

## Results

### Overview of ExoPLA

**Figure 1:**
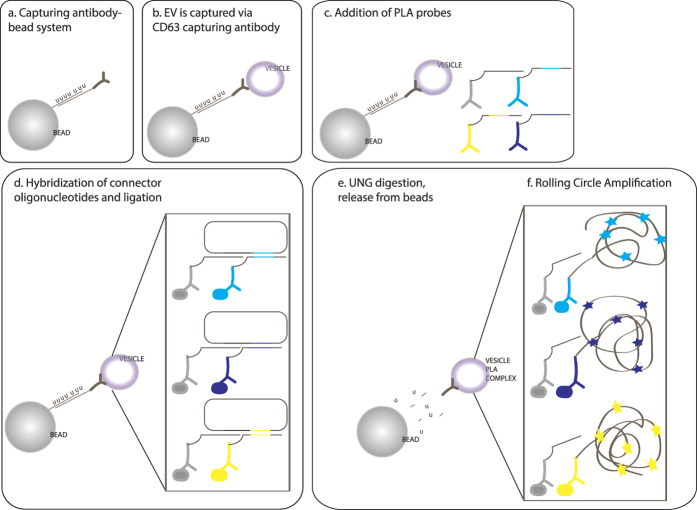
Schematic illustration of ExoPLA. Capturing antibodies are immobilized on streptavidin-modified beads via hybridization of uracil-containing oligonucleotides (**a**). The EVs are captured via CD63 antibodies (**b**) followed by addition of a set of PLA probes for each target of interest on the surface of the EV (**c**). The connector DNA oligonucleotides are hybridized to the DNA oligonucleotides on the antibodies allowing DNA circularization via enzymatic ligation (**d**). The vesicle-PLA complex is then released from the bead using enzymatic UNG digestion (**e**) followed by rolling circle amplification in solution (**f**). The detection signals from individual EVs are analyzed with a flow cytometer.

The ExoPLA technique uses capturing beads, allowing immobilized EVs to be stained through multiplex *in situ* PLA, for subsequent release before amplification and analysis of individual EVs by flow cytometry. Streptavidin-modified magnetic beads bind biotinylated oligonucleotides, which then immobilize oligonucleotide-conjugated capturing antibodies via hybridization (Fig. 1). The hybridization oligonucleotides contain uracil residues that can be enzymatically digested by uracil-DNA glycosylase (UNG) in order to release the captured EVs from the beads, before amplification of *in situ* PLA products via RCA and analysis by flow cytometry.

The multiplex *in situ* PLA^21^ that is the basis for the ExoPLA uses four PLA probes in order to create three differently colored signals. One of the PLA probes can pair with any of the other three to create three distinct circularized reporter DNA strands that are subsequently amplified and selectively detected (Fig. 1). We created a set of PLA probes targeting antigens present on most EVs. This common set of PLA probes included antibodies against dipeptidyl peptidase 4 (CD26), neprilysin (CD10), aminopeptidase N (CD13), and cathepsin B. Antibodies against CD63 were used for capturing the EVs and the other four were used as PLA probes, where CD26 could pair with any of the other three. We also created a set of PLA probes against selective markers, known only to be present on one of the EVs that we were investigating here (unpublished data). All probes and oligonucleotides are identified in Tables S1 and S2.

### Multicolor detection of EVs

**Figure 2:**
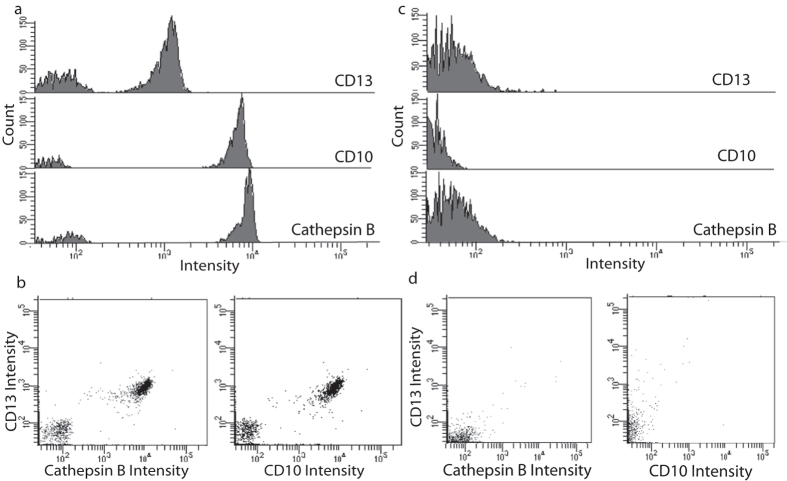
Detection of prostasomes using ExoPLA. Prostasomes diluted in buffer were detected with the common PLA probes, directed against, CD26, CD10, CD13 and Cathepsin B, using the BD Fortessa setting against FCS PMT. (**a**) Histograms of the three fluorophores, representing three different detected proteins. (**b**) Dot plots showing signals for EVs well over background, (**c,d**) illustrate the negative control (no prostasomes present) for the experiment shown in (**a,b**), respectively.

As a first attempt, we applied our ExoPLA approach using purified prostasomes as a model. These EVs are relatively well characterized with respect to proteins present on the vesicle surface^12^,^23^,^24^,^25^,^26^. We used the common set of PLA probes directed against CD26, CD10, CD13 and Cathepsin B, and the stained EVs were evaluated using a BD LSRFortessa or a BD LSR II flow cytometer (Fig. 2 and S2). Fluorescence triggering^27^,^28^ gave signals well over background, sufficient for further analyses. The results obtained by flow cytometry were also confirmed by analyzing the prostasomes using fluorescence microscopy demonstrating the multicolor probing and detection of these EVs (Fig. S1). The multicolor detection of EVs, as well as all other experiments described herein have been repeated at least three times.

### Performance of ExoPLA compared to a conventional bead-based assay

To investigate how ExoPLA compares with a commonly used bead assay, we chose the Exosome-Human CD63 Isolation/Detection kit from Invitrogen. The capturing target protein was the same – CD63 – and we used CD10 as detection target protein. In order to avoid any differences due to antibody preparations the same antibodies directed against CD10 were used in both assays. For the bead assay, we conjugated the detection antibody with fluorescein isothiocyanate (FITC), and for ExoPLA the detection oligonucleotide was similarly modified with FITC. Both assays were performed on the same serial dilution of EV samples. To investigate how well the ExoPLA assay performs using the common probes directed against CD10, CD26 and cathepsin B, we made a serial dilution of purified prostasomes in buffer and performed the analysis with all three sets of PLA probes for multicolor detection and only one pair of probes for single color detection.

**Figure 3:**
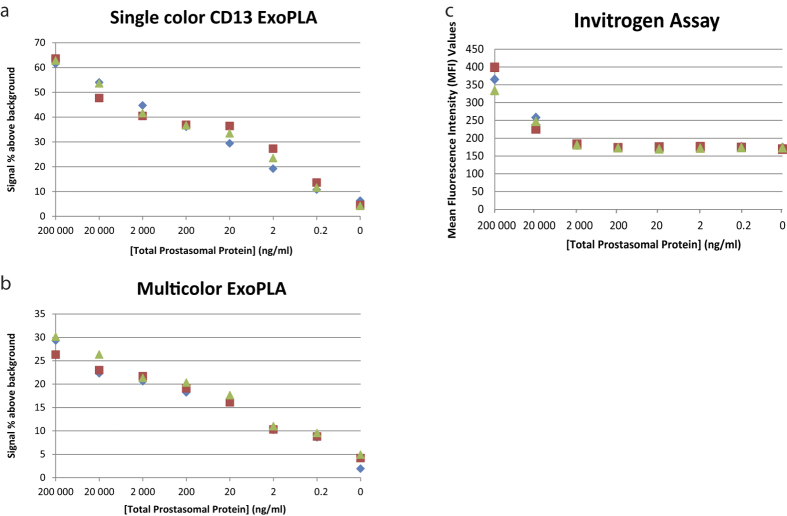
Performance of ExoPLA. The performance of single color- and multicolor ExoPLA was compared to the Invitrogen bead assay for EVs using serially diluted prostasomes in buffer. (**a**) and (**b**) are the titrations of single color ExoPLA and multicolor ExoPLA, respectively, where Y-axis shows percent positive events above background (percent of parent). A gate was placed in the plots for the events that were above the background and counted as positives. (**c**) shows the Invitrogen bead assay titration were the Y-axis represents the mean fluorescence intensity (MFI obtained from BD DIVA software) of the beads. The x-axis indicates the concentration of prostasomes in all three plots. The red, green and blue curves in each plot represent three independent experiments.

The starting concentration of total protein from prostasomes was 200 μg/ml serially diluted in 10-fold steps. Results for the flow cytometric data from the Invitrogen assay were reported in Mean Fluorescence Intensity (MFI) values per bead, while the ExoPLA method yielded numbers of events, corresponding to individual detected EVs. The results were normalized against background and compared (Fig. 3). The ExoPLA method values allowed detection of as little as 200 pg/ml total prostasome protein concentration, compared to 20 μg/ml for the commercial bead-based assay. In addition, the ExoPLA protocol is more rapid, since it can be carried out in a single day compared to the two-days protocol for the commercial bead-based assay, as the latter protocol requires overnight incubation of the EVs on the beads.

### ExoPLA can distinguish different populations of EVs

Prostasomes express a selective marker, the membrane glycoprotein Thy-1, which distinguishes them from other EVs (unpublished data). Similarly, EVs isolated from the cell line U937 express the granulocyte colony-stimulating factor receptor (CD114), which is not present on prostasomes or other EVs used in these experiments (unpublished data).

**Figure 4:**
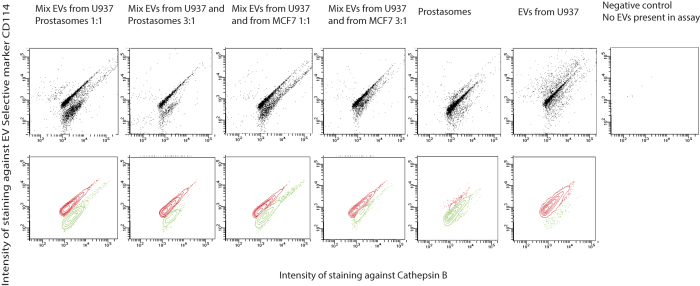
Selective detection of EVs. Detection of EVs isolated from U937 cells and prostasomes separately or mixed at ratios of 1:1, and 3:1, and mixed EVs from U937 cells and MCF7 cells at ratios of 1:1, and 3:1, using multicolor ExoPLA where one of the four PLA probes is the selective PLA probe against CD114, only present on EVs from U937 cells, in combination with common probes CD13 and Cathepsin B. The ratios are based on the total protein concentrations for the EVs. The top row displays all events and the bottom row illustrates gated plots. In the gated, bottom row plots the red populations represents EVs from U937, positive for CD114, and the green populations are representing prostasomes or EVs from MCF7, negative for CD114. The far right plot represents a negative control where no EVs were present during the assay.

In order to examine whether ExoPLA can be utilized to distinguish different populations of EVs in a sample, EVs isolated from the myeloid cell line U937 were mixed either with EVs isolated from the breast cancer cell line MCF7 or with prostasomes isolated from seminal fluids, at a ratio of 1:1 or 3:1 based on total protein concentrations. The sources of EVs were selected to represent tissues prone to developing different forms of cancer. The EVs from MCF7 and the prostasomes, lacking CD114 (unpublished data), were used as unspecific controls displaying the common markers. Antibodies against CD63 were used to capture all EVs. Antibodies directed against CD114 were used to prepare PLA probes selective for EVs isolated from U937 cells. For selective detection of EVs from U937 cells we used PLA probes against CD114 together with probes against CD13, CD26 and cathepsin B. The CD114 selective probes could clearly identify and distinguish EVs from the U937 cell line both when mixed with prostasomes and with EVs from the MCF7 cell line (Fig. 4 and S3).

### ExoPLA can enumerate EVs in the complex matrix of human plasma

**Figure 5:**
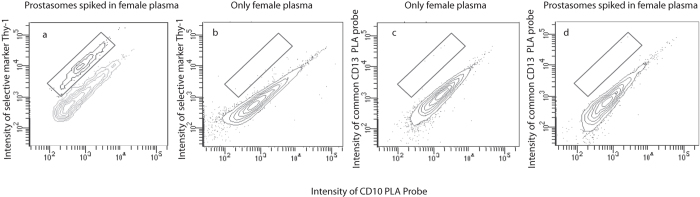
Detection of EVs spiked in plasma. To investigate ExoPLA performance in a complex matrix, 10 μg total protein of prostasomes was spiked in 10% female blood plasma. (**a**) ExoPLA was performed with a selective probe detecting Thy-1 and common probes CD26, CD10 and Cathepsin B present on prostasomes. (**b**) As control female plasma with no spiked-in prostasomes was assessed with the selective and the common probes. (**c**) female plasma assessed with only common probes, where the selective Thy-1 probe was replaced with CD13. (**d**) The same matrix with spiked-in prostasomes was tested with ExoPLA using common probes. The population with Thy-1 positive EVs was only observed in samples spiked with prostasomes.

To investigate the performance of the ExoPLA assay in a complex biological matrix, prostasomes at concentrations corresponding to 10 μg or 200 μg/ml total protein concentrations were spiked into 10% human plasma from a healthy female. We performed ExoPLA with the selective set of PLA probes, including the probe directed against Thy-1. As controls, we also performed ExoPLA with the common set of probes in 10% female plasma with or without spiked-in prostasomes. Assays including the selective probes clearly distinguished the population of prostasomes spiked into female plasma from endogenous EVs present in female plasma. This separate population was not observed in control experiments without spiked-in prostasomes or when a common set of probes were used (Fig. 5 and S4).

## Discussion

The realization that EVs play a role in homeostasis and disease, highlights the need for rapid, sensitive and specific methods to measure them. Flow cytometry is a widely used method for routine quantification and qualification of cells, and it would be beneficial if EVs could be analyzed in a similar manner. However, this has proven challenging due to their small sizes and low number of surface proteins. To circumvent these obstacles more sophisticated flow cytometers can be used that can detect smaller particles^29^. Other approaches use bead-based methods or membrane dyes to detect and examine bulk levels of EVs present in a sample. By using *in situ* PLA we have now established a protocol to detect individual EVs with signals well above the background using conventional flow cytometers through ExoPLA. Two advantageous features of ExoPLA are i) the signal amplification that ensures that recognition of a small numbers of surface molecules suffices to produce prominent fluorescent signals for each EV, and ii) the ability to monitor individual EVs for the presence of several proteins, which enables identification and distinction of EVs that may share several proteins on their surfaces. The ability to investigate EVs one by one instead of through bulk measurements provides a means to investigate heterogeneous populations of EVs, even in a complex matrix such as plasma, also enabling downstream analysis of sorted EVs.

The application of ExoPLA on a serial dilution of EVs revealed that the sensitivity of the method greatly surpasses that of a commonly used bead-based assay by detecting EVs at very low concentrations. For the serial dilution experiments we observed a slightly higher number of detected EVs through single color ExoPLA than for the multicolor PLA. This is a likely consequence of the fact that in the latter assays one PLA probe must cooperate with each of three other PLA probes to give rise to the three classes of RCA products detected in separate colors. In order for an EV to be detected, all three fluorescence intensities must exceed a threshold, set by the gating, and a lower intensity in one of the channels will place some of the EVs below the threshold. ExoPLA was shown to selectively detect male prostasomes spiked in female blood plasma demonstrating the method’s high specificity due to its requirement for recognition by up to five antibodies.

EVs are assuming rapidly increasing interest in biomarker analysis because of their presence in most bodily fluids, and the circumstance that they may harbor proteins and transcripts that can reveal the identity of the originating tissue or perhaps tumor^22^,^30^,^31^. EVs from different tissues may differ according to the presence of proteins specific for the cells from which they are released. These selective marker proteins may be used in ExoPLA to identify the source of EVs in bodily fluids, and potentially to use them as biomarkers for disease. Nevertheless, selection and evaluation of proper affinity reagents for capture and detection of particular EVs will be of great importance for development of any specific assay. Accordingly, the ExoPLA method presented here opens up for a more informative, reliable and robust means to routinely characterize and quantify EVs using flow cytometry, distinguishing their tissues of origin. Future studies of patient samples compared to gender and age-matched controls will reveal the diagnostic and prognostic potential of this technology.

## Materials and Methods

### Antibodies for capturing and detection of EVs

Purified mouse anti-human CD63 antibodies were purchased from BD Biosciences. Polyclonal goat anti-human cathepsin B, monoclonal rat anti-human DPPIV/CD26, polyclonal goat anti-human neprilysin/CD10, polyclonal goat anti-human G-CSF R/CD114, and polyclonal sheep anti-human CD90/Thy1 antibodies were all purchased from RnD Systems. Monoclonal mouse anti-human CD13 antibodies were purchased from AbD Serotec.

### Covalently coupling of DNA oligonucleotides and fluorophores to antibodies

For multicolor detection of EVs one PLA probe (Table S1) was prepared for each target protein by covalently attaching DNA oligonucleotides to specific antibodies. The conjugations of azide-modified DNA oligonucleotides to the antibodies were performed using copper-free click chemistry^32^,^33^. A bifunctional chemical crosslinker (dibenzylcyclooctyne-NHS (DBCO-NHS), Jena Biosciences or Sigma Aldrich) was used to activate the antibody according to the manufacturers’ instructions, modified as follows: 100 μg of antibody at a concentration of 2 mg/ml was used for each conjugation. After incubating the antibody with 360 μM DBCO-NHS ester for 30 min at room temperature (RT) the reaction was stopped by adding excess amino groups in the form of 1 M Tris-HCl pH 8.0. Any DBCO not coupled to the antibodies was removed by Zeba spin desalting columns 40 K MWCO (Thermo Scientific). Next, azide modified oligonucleotides were added to the antibodies modified with crosslinker at 2.5 x molar excess and incubated over night at 4 °C. Sodium azide (Sigma) was added to a final concentration of 0.05% and the PLA probes were kept at 4 °C. Polyclonal goat anti-human neprilysin/CD10 antibodies were labeled with NHS-fluorescein (Thermo Scientific Cat. No. 46409) according to the manufacturer’s protocol.

### Cell lines and isolation of EVs

The U937 cell line was grown in RPMI 1640 media (Sigma-Aldrich) supplemented with 10% fetal bovine serum (FBS), 2 mM L-glutamine and 100 U/ml penicillin-streptomycin. The MCF7 cells were grown in DMEM media (Sigma-Aldrich) supplemented with 10% FBS, 1% L-glutamine and 1% penicillin-streptomycin. The cell lines are commercially available from ATCC (U937 ATCC# CRL-1593.2 & MCF7 ATCC# HBT-22). MCF7 and U937 cells were grown to 75% confluence without FBS for 48 h prior to EV isolation. U937 cells were centrifuged 3 min at 1,200 × g to recover a cell free supernatant. Supernatants from cultures of both cell lines were used for isolation of EVs, and protease inhibitors were added (Complete Mini, Roche). The cell free supernatants were then sequentially centrifuged at 3,000 × g for 10 min and at 10,000 × g for 10 min, filtered through a 0.45 μm PES filter (VWR) and then finally centrifuged at 100,000 × g for 2 h, followed by one washing step and a second centrifugation at 100,000 × g for 2 h. Pelleted EVs were resuspended in a small amount of sterile filtered PBS supplemented with protease inhibitors (Complete Mini, Roche). Samples were stored at −80 °C and later prepared for further analysis.

### Purification of prostasomes

Human seminal plasma was obtained from Uppsala University Hospital, collected according to established routines^34^ and kept at −20 °C. Thawed seminal plasma was centrifuged for 10 min at 4 °C and 3,000 × g, and the decanted supernatant was centrifuged for 30 min at 4 °C and 10,000 × g to eliminate possible cell debris and larger vesicles. Next, the resulting supernatant was subjected to ultracentrifugation for 2 h at 4 °C and 100,000 × g using Rotor 90 Ti (Beckman Coulter, Brea, CA, USA)^23^. The pellet was resuspended in phosphate buffered saline (PBS) pH 7.6, and loaded onto an XK16/70 Superdex 200 gel column (GE Healthcare, Uppsala, Sweden)^35^. Fractions were collected at a flow rate of 5 ml/h and the absorbance at 260 nm (nucleic acid) and 280 nm (proteins, indicating prostasome presence) was measured. Fractions with elevated absorbance were pooled and ultracentrifuged for 2 h at 4 °C and 100,000 × g. The pellets were resuspended in PBS and floated onto a gradient of 1 M, 1.5 M and 2 M sucrose and ultracentrifuged for 20 h at 4 °C and 85,000 × g using an SW28.1 rotor (Beckman Coulter). The main fraction at 1.5 M (density range 1.13–1.19 g/ml) was pelleted by ultracentrifugation for 2 h at 4 °C and 100,000 × g. The pellet was resuspended, collected and adjusted to a protein concentration of 2 mg/ml using a BCA protein assay kit (Merck-Millipore, Darmstadt, Germany). Prepared prostasomes were kept at −70 °C until use. All experiments were approved by the Ethical Committee of Uppsala University in accordance with ethical standards and the Declaration of Helsinki, and samples were obtained after informed consent. Prostasomes were identified and characterized in scanning electron microscopy (SEM)^36^. 2% glutaraldehyde in PBS was used to fixate the prostasome pellets. The pellets were washed in distilled water and then to an ethanol series, with 70% EtOH for 10 min, 95% EtOH for 10 min and 99.9% EtOH for 15 min, performed at 4 °C. The pellets were placed in acetone before drying in a critical point dryer (Balzer, CPD 010, Lichtenstein) using carbon dioxide. Mounting was performed on separate aluminum stubs and the pellets were coated with Carbon (Bal-Tec MED 010, Lichtenstein). An Ultra 55 field emission scanning electron microscope (Zeiss, Oberkochen, Germany) at 5 kV was used for analyses (Fig. S5).

### Immobilization of antibodies on microparticles

200 μl (10 mg/ml) T1 Dynabeads Magnetic Beads (Life Technologies) were washed twice using 1 x phosphate buffered saline (PBS) with 0.05% Tween 20 (Sigma-Aldrich) (PBST). 50 nM conjugated CD63 antibodies, carrying oligonucleotide nr 13 (Table S2) and 300 nM biotinylated oligonucleotide nr 14 (Table S2) were mixed in 1 x PBS supplemented with 1% BSA (New England Biolabs) (PBSB) and added to the beads, followed by incubation for 1 h at RT. The beads were washed twice with PBST and reconstituted in 400 μl PBST.

### *In situ* PLA

For each *in situ* PLA reaction 200 μg/ml EVs were incubated with 1 μl oligonucleotide-coated beads in Odyssey blocking buffer (LI-COR Biosciences) to a final volume of 50 μl, and incubated for 60 min at 37 °C with rotation. Washing with 1 x Tris-buffered saline (TBS) with 0.05% Tween20 (Sigma-Aldrich) (TBST) was followed by the addition of PLA probes. All PLA probes were diluted to 1:50 from the previous prepared conjugations, in TBST with 5% sterile filtered goat serum and 2.6 pg/μl sonicated salmon sperm DNA, and incubated for 60 min at 37 °C with rotation. After three washes with TBST, the mixture was incubated with the two circularization DNA oligonucleotides at 125 nM in ligation buffer (20 mM Tris acetate pH 7.5, 20 mM magnesium acetate, 100 mM potassium acetate, 25 mM NaCl, 0.25 μg/μl BSA (New England Biolabs), 0.05% Tween 20, 1.0 mM ATP (Fermentas) with 0.04 U/μl T4 DNA ligase (Fermentas)) for 30 min at 37 °C with rotation. After washing with TBST the beads were incubated for 30 min at 37 °C with rotation for UNG digestion in 0.05 U/μl UNG (Fermentas) in 1 x UNG buffer (Fermentas) with 0.5 μg/μl BSA (New England Biolabs) to release captured EVs. The subsequent RCA reaction and labeling of PLA reaction products on the released EVs was performed in the supernatant after the UNG digestion. 1 U/μl phi-29 DNA polymerase (Fermentas) and 10 nM fluorophore-labeled specific tag oligonucleotides were mixed with RCA buffer (2 x phi-29 DNA polymerase buffer (Fermentas) with 0.5 μg/μl BSA, 0.375 mM dNTP (Thermo Scientific), pH 7.5)) and incubated for 90 min at 37 °C. The samples were diluted in 0.2 μm filtered PBS after the last step of the assay. The signals were detected with BD Fortessa or BD LSRII flow cytometers using either the photomultiplier tube option for the forward scatter channel (FCS PMT), only forward scatter/side scatter (FCS/SSC), or fluorescence triggering and the threshold was set for all colors at 750 using 0.2 μm filtered PBS.

In the titration trial the purified prostasomes were serially diluted in Odyssey blocking buffer PBS from LI-COR, with a starting concentration of total prostasomal protein of 200 μg/ml in 10-fold steps.

### Invitrogen bead assay

The assay was performed according to the manufacturer’s protocol. Five μl neprilysin/CD10 antibodies labeled with NHS-fluorescein was added to the beads to a final volume of 100 μl and incubated for 30 min at 37 °C. The beads were then washed once in PBS and resuspended in 200 μl PBS prior to analysis.

### Flow cytometry and data analysis

All flow cytometry analyses were performed either on a BD LSRFortessa or a BD LSR II (BD Biosciences). Fluorescence triggering was used in all experiments, except when we wanted to display the background where FCS-PMT, or FCS/SSC was used. For the titration-assays, for the ExoPLA samples, a gate for the positive events was placed after the recording of events from a sample of EVs in filtered (0.22 μm) PBS, and the threshold was set at 750 for all the fluorophores. All the events that were recorded in this gate were regarded as positives. The number of events for each sample were counted during one minute.

Data analysis was performed using BD FACSDiva software version 8.0 (BD Biosciences). For the titration-assays the results using the Invitrogen assay were reported in Mean Fluorescence Intensity (MFI) values per bead for each dilution step. For the ExoPLA method the number of events over background, corresponding to detected EVs, was recorded for each dilution step.

## Additional Information

**How to cite this article**: Löf, L. *et al*. Detecting individual extracellular vesicles using a multicolor *in situ* proximity ligation assay with flow cytometric readout. *Sci. Rep*. **6**, 34358; doi: 10.1038/srep34358 (2016).

## Supplementary Material

Supplementary Information
