# Supplementary material for: Detecting individual extracellular vesicles using a multicolor in situ proximity ligation assay with flow cytometric readout
Source: Sci Rep. 2016 Sep 29;6:34358. doi: 10.1038/srep34358 (PMC5041182; doi:10.1038/srep34358)
Supplement: Supplementary Information [file srep34358-s1.pdf]

Supplementary data

Detecting individual extracellular vesicles using a multicolor *in situ* proximity ligation assay with flow cytometric readout

Liza Löf[a], Tonge Ebai[a], Louise Dubois[b], Lotta Wik[a], K. Göran Ronquist[b], Olivia Nolander[a], Emma Lundin[a], Ola Söderberg[a], Ulf Landegren[a] and Masood Kamali-Moghaddam[a*]

[a]Department of Immunology, Genetics & Pathology, Science for Life Laboratory, Uppsala University, SE-751 08 Uppsala, Sweden.

[b]Department of Medical Sciences, Clinical Chemistry, Uppsala University, SE-751 85 Uppsala, Sweden

[*]Corresponding author: Department of Immunology, Genetics & Pathology, Science for Life Laboratory, Uppsala University, SE-751 08 Uppsala, Sweden.

Telephone: +46-70 7454366

Email: masood.kamali@igp.uu.se

Table S1. Description of antigen targets and their antibodies

Target / Antibody	Usage. Conjugate	Extracellular vesicle, type of marker
**CD63**	Capturing. Coupled to oligonucleotide for release	All, common marker
**Dipeptidyl peptidase 4 (CD26)**	Coupled to oligonucleotide 1	All, common marker
**Neprilysin (CD10)**	Coupled to oligonucleotide 2	All, common marker
**Aminopeptidase N (CD13)**	Coupled to oligonucleotide 3	All, common marker
**Cathepsin B**	Coupled to oligonucleotide 4	All, common marker
**Thy-1 membrane glycoprotein (Thy-1)**	Coupled to oligonucleotide 3	Prostasomes, selective marker
**Granulocyte colony-stimulating factor receptor (CD114)**	Coupled to oligonucleotide 2	Exosomes from cell line U937, selective marker

Table S2. Oligonucleotides and antibodies used in capturing, release via UNG digestion, and *in situ* PLA.

Oligonucleotide	Description	DNA sequence
1	CD26 general PLA probe oligonucleotide	5’: Azide GACGCTAATAGTTAAGACGCTT
2	CD10/ CD114 PLA probe oligonucleotide	5’ Azide: AAAAAAAAAATATGACAGAACATACGGTCTCGCAGATCGCTTAGACACTCTT
3	CD13/Thy1 PLA probe oligonucleotide	5’ Azide: AAAAAAAAAATATGACAGAACGGACGATCATCCAGCACTAGTAGACACTCTT
4	Cathepsin B PLA probe oligonucleotide	5’ Azide: AAAAAAAAAATATGACAGAACCGGGCGACATAAGCAGATACTAGACACTCTT
5	Tag-specific oligonucleotide for CD10/CD114	5’phosphate: AGCGATCTGCGAGACCGTAT
6	Tag-specific oligonucleotide for CD13&Thy1	5’phosphate: CTAGTGCTGGATGATCGTCC
7	Tag-specific oligonucleotide for Cathepsin B	5’phosphate: GTATCTGCTTATGTCGCCCG
8	Circulation oligonucleotide short	5’phosphate: GTTCTGTCATATTTAAGCGTCTTAA
9	Circulation oligonucleotide long	5’phosphate: CTATTAGCGTCCAGTGAATGCGAGTCCGTCTAAGAGAGTAGTACAGCAGCCGTCAAGAGT GTCTA
10	Tag-specific detection oligonucleotide for CD10/CD114	5’-Cy5: AGCGATCTGCGAGACCGTATUUUU
11	Tag-specific detection oligonucleotide for CD13/Thy1	5’-Pacific Blue:CTAGTGCTGGATGATCGTCCUUUU
12	Tag-specific detection oligonucleotide for Cathepsin B	5’-Cy3: GTATCTGCTTATGTCGCCCGUUUU

**13**	Release UNG digestion oligonucleotide/ CD63 capturing oligonucleotide	5´Azide:AAAAACGAUUCGAGAACGUGACUGCCAUGCCAGCUCGUACUAUCGAATAATC GTACCCT
**14**	Release UNG digestion oligonucleotide	5’Biotin: CGAUAGUACGAGCUGGCAUGGCAGUCACGUUCUCGAAUCGUUUU

a

b

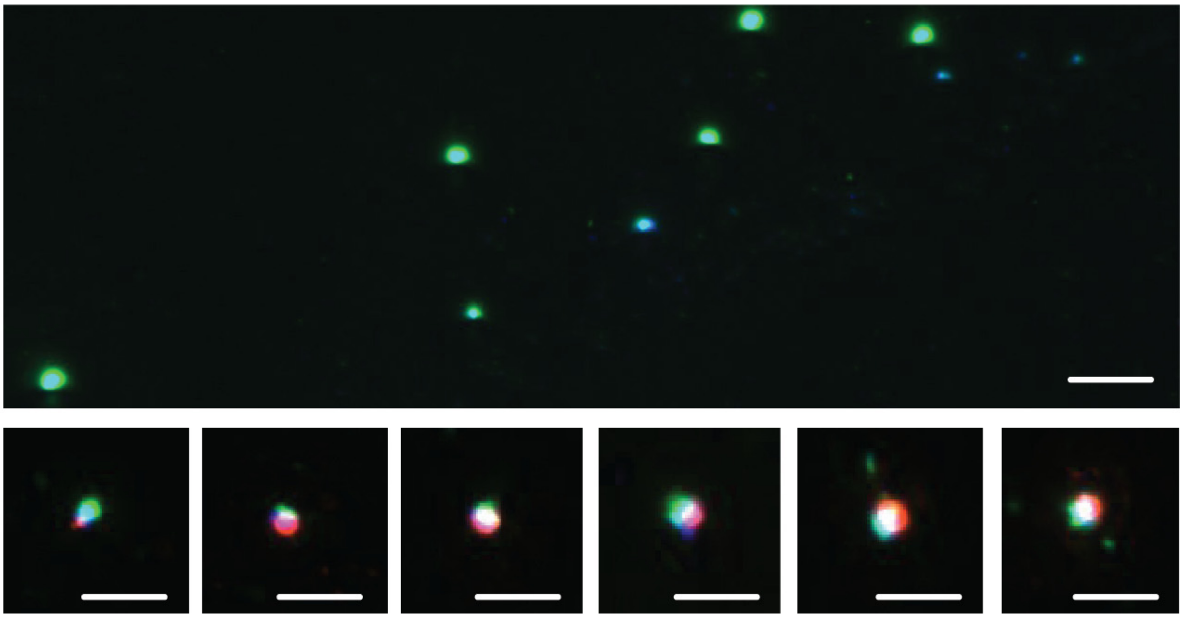

Figure S1. Multicolor detection of EVs using fluorescence microscopy. To confirm the data from multicolor ExoPLA as analyzed through flow cytometry, the probed prostasomes were also analyzed by fluorescence microscopy. a) represents results from, a dual color assay, were also some background from fluorophores can be seen and b) represents triple color detection as used in figure 2. Scale bar represents 5 µm.

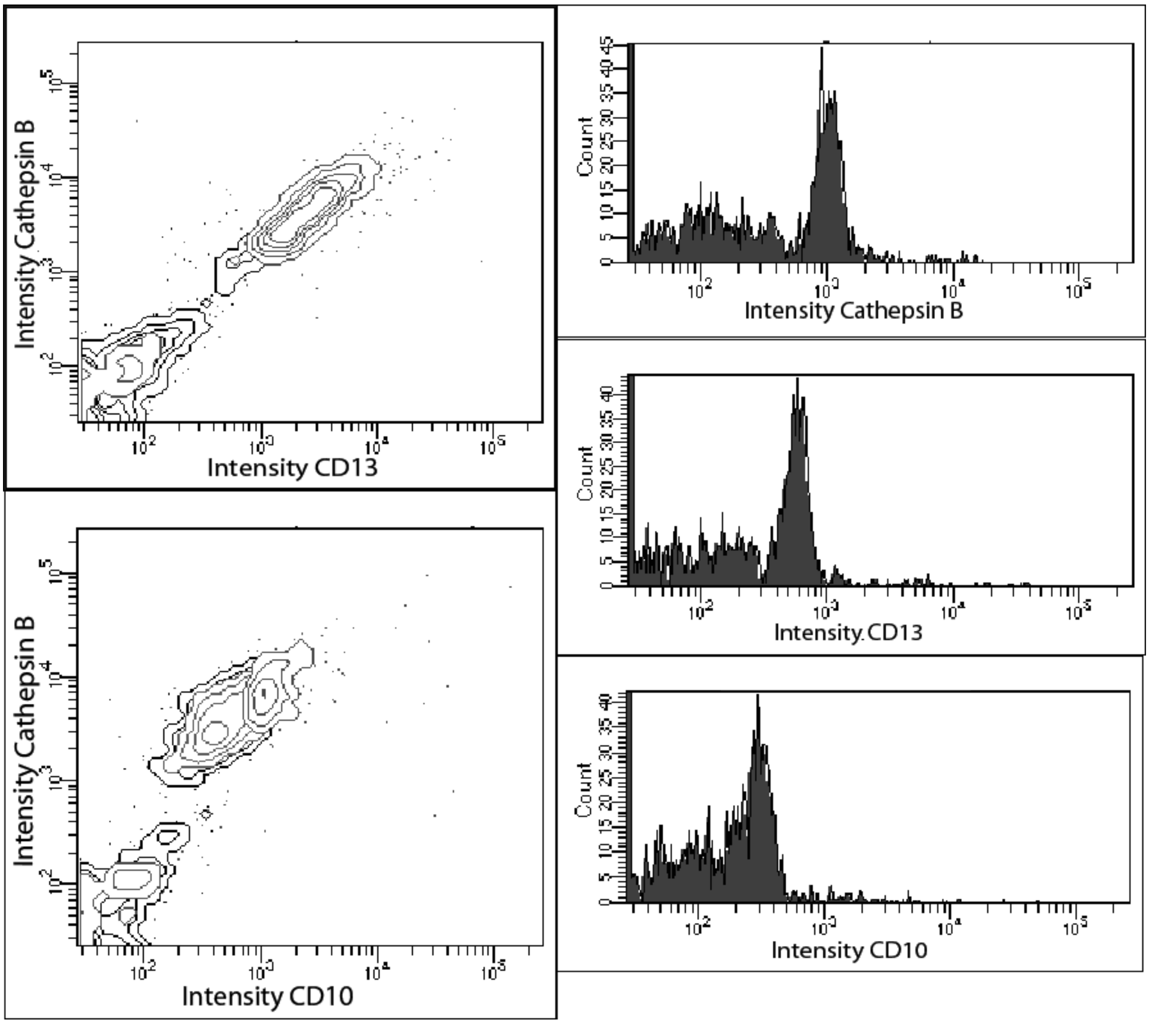

Figure S2. Replicate experiments demonstrating multiplex detection of prostasomes using ExoPLA. Prostasomes diluted in buffer were detected with the common PLA probes, directed against, CD13, CD10 and Cathepsin B, using the BD Fortessa setting against FCS PMT. Histograms of the three fluorophores, representing three different detected proteins. Dot plots showing signals for EVs over background.

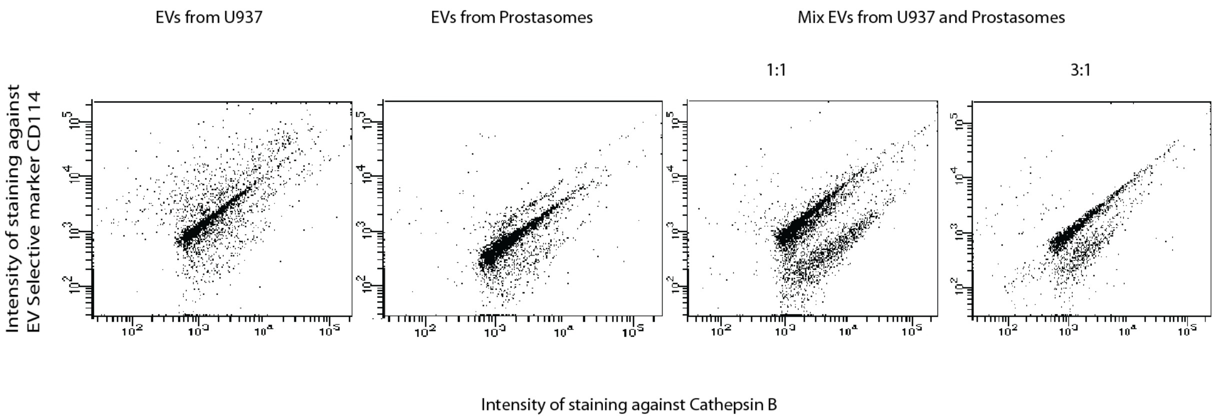

Figure S3: Replicate experiments of detection of mixed EVs. Detection of EVs isolated from U937 cells and prostasomes separately or mixed at ratios of 1:1, and 3:1.Using multicolor ExoPLA, where one of the three PLA probes is the selective PLA probe against CD114, only present on EVs from U937 cells. The ratios are based on the total protein concentrations for the EVs.

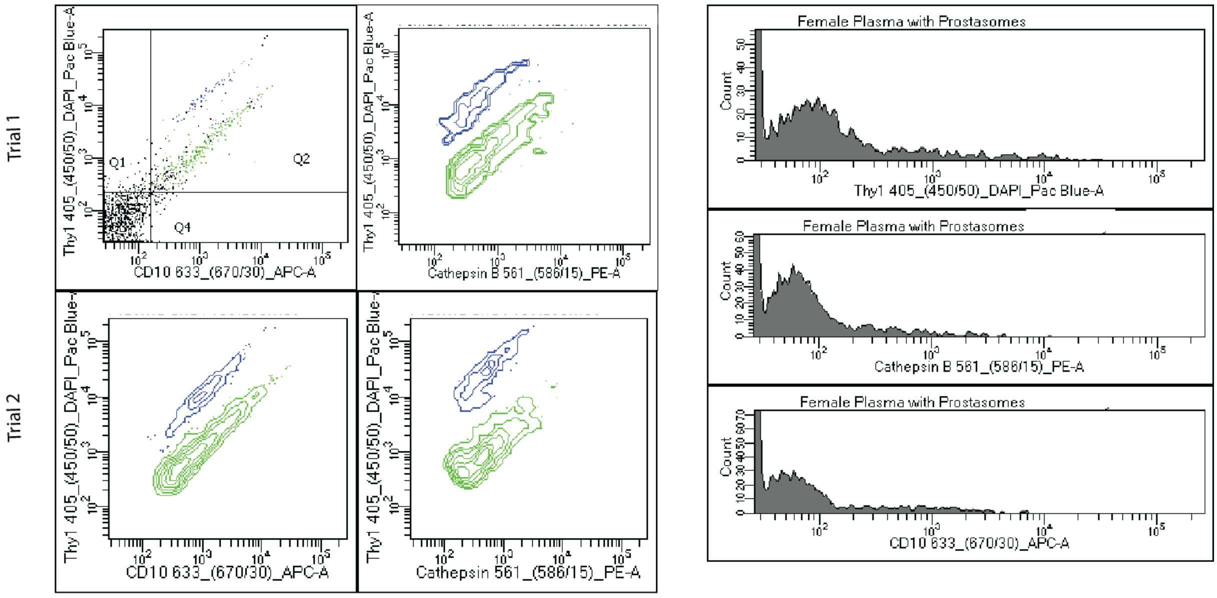

Figure S4. 2 Replicate experiments for detection of EVs spiked in plasma. To investigate ExoPLA performance in a complex matrix, 10 µg total protein of prostasomes was spiked in 10% female blood plasma. ExoPLA was performed with selective probes detecting Thy-1, present on prostasomes. On the left hand side 2 dot plots are shown and on the right hand side the histograms for trial 1 is shown. Trial 1 and 2 are replicated of each other.

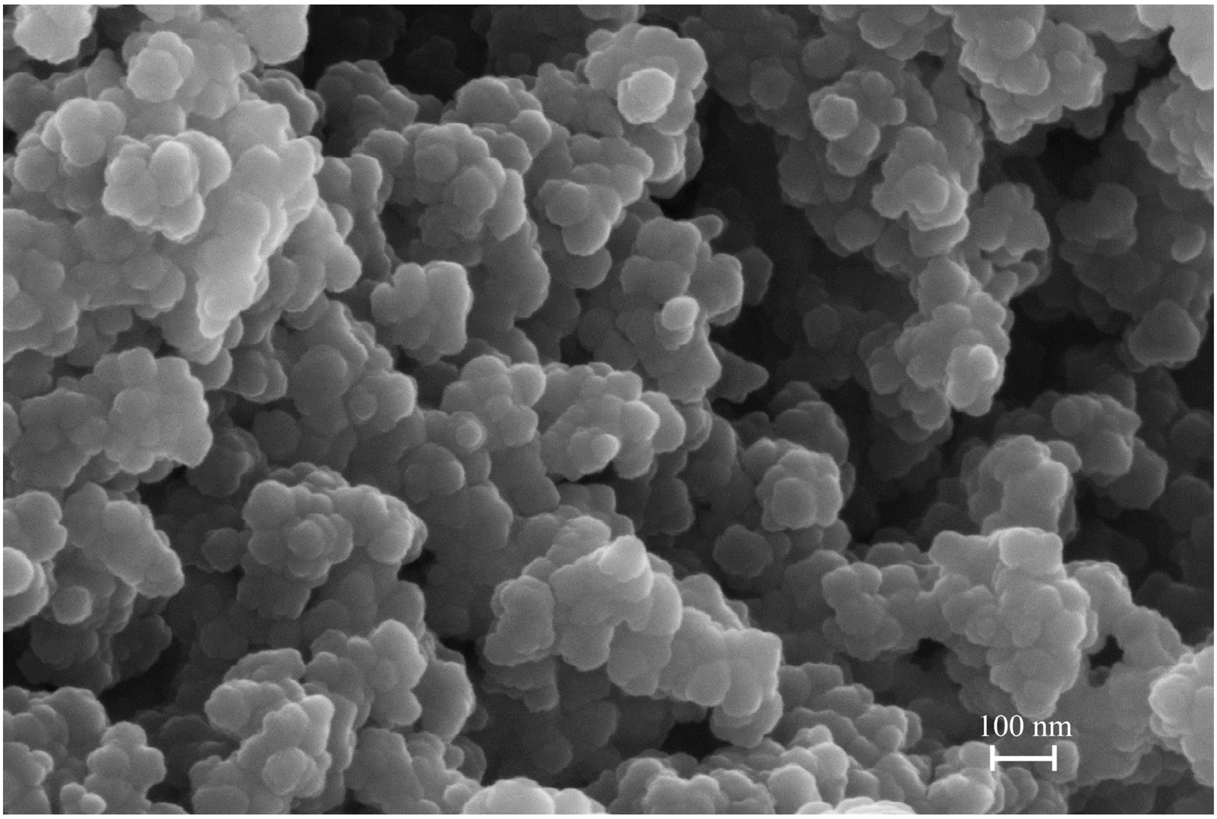

Figure S5. SEM image of the prostasomes, with diameters of approximately 100 nm and a rounded appearance.

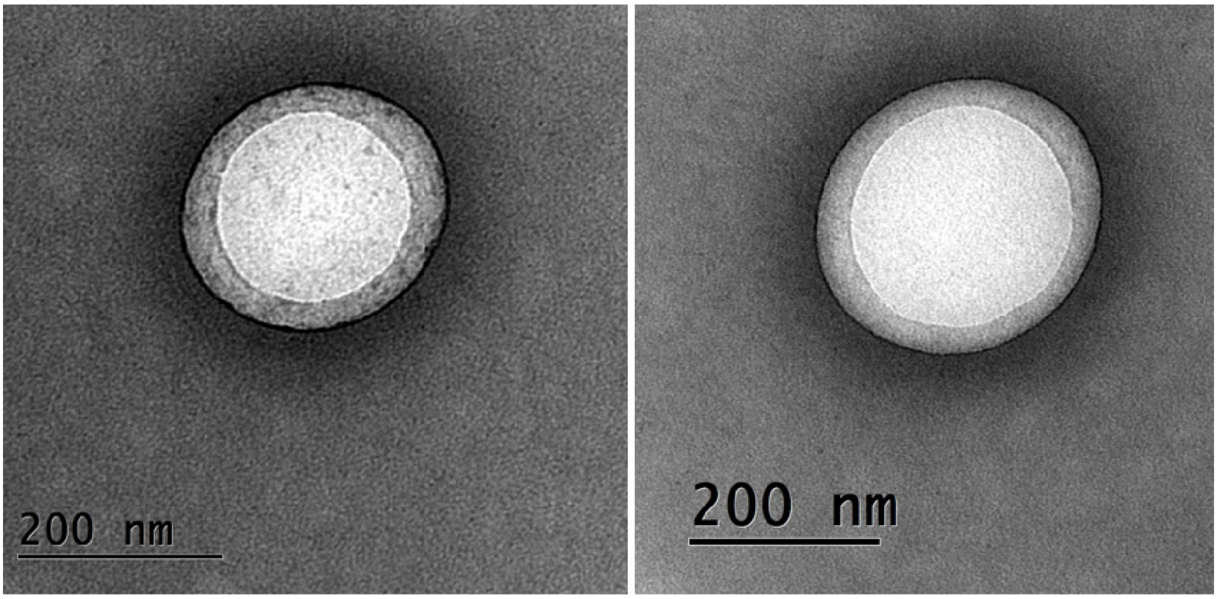

Figure S6. TEM image of the MCF7 EVs. These EVs also show a globular appearance and the lipid bilayer is visualized.
